# Potential Applications and Risks of Supranutritional Selenium Supplementation in Metabolic Dysfunction-Associated Steatotic Liver Disease: A Critical Review

**DOI:** 10.3390/nu17152484

**Published:** 2025-07-30

**Authors:** Chuanming Liu, Ke Chen, Zijian Xu, Lianshun Wang, Yinhua Zhu, Zhengquan Yu, Tong Li, Jiaqiang Huang

**Affiliations:** 1 Key Laboratory of Precision Nutrition and Food Quality, Department of Nutrition and Health, China Agricultural University, Beijing 100193, China; zb20223311105@cau.edu.cn (C.L.); xuzijian@cau.edu.cn (Z.X.); zhuyinhua@cau.edu.cn (Y.Z.); 2 Beijing Laboratory of Food Quality and Safety, Department of Nutrition and Health, China Agricultural University, Beijing 100193, China; 3 College of Veterinary Medicine, China Agricultural University, Beijing 100193, China; b20243050504@cau.edu.cn; 4 College of Fisheries and Life Science, Dalian Ocean University, Dalian 116023, China; wanglianshun@dlou.edu.cn; 5 College of Biological Sciences, China Agricultural University, Beijing 100193, China; zyu@cau.edu.cn; 6 Institute of Agricultural Products Preservation and Processing Technology (National Engineering and Technology Research Center for Preservation of Agricultural Products), Tianjin Academy of Agricultural Sciences, Key Laboratory of Storage and Preservation of Agricultural Products, Ministry of Agriculture and Rural Affairs, Tianjin 300384, China

**Keywords:** metabolic dysfunction-associated steatotic liver disease, non-alcoholic fatty liver disease, selenium, supranutritional, selenoproteins, selenium nanoparticles

## Abstract

Metabolic dysfunction-associated steatotic liver disease (MASLD) is one of the most prevalent chronic diseases in the world, lacking specific pharmacological interventions or well-established treatments. MASLD involves intricate pathological mechanisms characterized by oxidative stress and robust inflammatory responses. Selenium, an essential trace element, plays a critical role in antioxidation, regulation of inflammation, anticancer activity, and so on. Recent studies have reported that supplementation with selenium could alleviate MASLD and associated hepatic disorders, while excessive consumption may result in insulin resistance or even selenosis. Therefore, supranutritional selenium supplementation can be more suitable for the therapy and prevention of MASLD. This paper comprehensively reviews research about selenium and MASLD to highlight the potential applications and risks of supranutritional selenium supplementation in MASLD, following three steps: conducting a search, reviewing research articles and reviews, and discussing results. The keywords for the search include but are not limited to selenium, MASLD, supranutritional, hepatic diseases, selenoproteions, and selenium nanoparticles (SeNPs). We have reached the following conclusions: supranutritional selenium supplementation exhibits promising potential as a strategy to treat MASLD, but there are still some risks, depending on the dose and form of selenium; evaluating MASLD severity and selenium nutritional status accurately, as well as supplementing with superior forms of selenium (e.g., organic selenium and SeNPs), can further ensure the safety and efficacy of selenium supplementation. However, relationships between selenium homeostasis disorders and the occurrence and development of MASLD have not been fully elucidated. Methods for comprehensively assessing selenium status and mechanisms of selenosis require further investigation and research.

## 1. Introduction

Non-alcoholic fatty liver disease (NAFLD) refers to a series of diseases characterized by excessive accumulation of triglycerides (TGs) in hepatocytes without substantial alcohol stimulation or viral invasion. NAFLD is one of the most common chronic liver diseases in the world, and its global prevalence is around 25%, reaching up to 90% in morbidly obese people [1]. What is even worse, NAFLD cases are forecasted to increase by approximately 21% worldwide from 83.1 million (2015) to 100.9 million (2030) [2]. Consequently, the outlook given the formidable challenge posed by NAFLD appears far from optimistic. In June 2023, to better describe what chronic liver disease is rather than what it is not and to reduce potential stigma, a new term, “metabolic dysfunction-associated steatotic liver disease (MASLD)”, was published to replace the term “NAFLD” [3]. The latest research has provided evidence that data generated in the past three decades for NAFLD can be used interchangeably for MASLD [4]. In this review, we also use the new terminology “MASLD” instead of “NAFLD”. The occurrence and development of MASLD may be associated with abnormal lipid metabolism, oxidative stress, mitochondrial dysfunction, and other factors [5], but the specific mechanisms remain unclear. There is currently no special medicine for MASLD, and most of the traditional medicines for obesity have undesirable side effects [6]. Balanced diets and moderate exercise are effective measures to improve MASLD, but their effects depend on the compliance of patients. Consequently, safer and more effective therapeutic and preventive approaches for MASLD urgently need to be explored.

Selenium is an essential trace element in the human body and plays an important role in regulatory and metabolic functions, such as antioxidation, regulation of inflammation, and anticancer activity [7]. Selenium effectively mitigates oxidative stress-induced damage to cells, tissues, and organs [8]; modulates the expression of pro-inflammatory factors and anti-inflammatory factors [9]; and enhances immune cell activities [10], thereby maintaining normal physiological functions. Based on these aforementioned effects, selenium supplementation has the potential for therapeutic interventions and preventive applications in MASLD [11,12]. The majority of selenium intake comes from dietary supplements, with the human body absorbing approximately 80% of it [13]. Animal liver, animal kidneys, seafood, and cereals are all excellent food sources of selenium [7]. Selenium is very unequally distributed worldwide, and many countries suffer from selenium deficiency, including the United Kingdom, France, Germany, Spain, the Netherlands, China, and India [14,15]. People residing in these deficient areas particularly require selenium supplementation since long-term selenium deficiency may increase the risk of diseases such as Keshan disease, Kaschin–Beck disease, and cardiovascular disease [16]. Table 1 summarizes dietary reference selenium intakes for adults in different countries or bodies. Compared with other trace elements, the range of selenium dosages from deficiency to toxicity is relatively narrow. Selenium excess can cause insulin resistance (IR), which is closely related to diabetes and MASLD [17,18,19], or exert some toxic symptoms which are collectively called “selenosis”. Selenosis is even worse in populations suffering from food and nutritional insecurity and those who are underweight or malnourished, who often already have hepatomegaly. In addition, different forms of selenium, such as sodium selenite, selenomethionine (SeMet), and selenium nanoparticles (SeNPs), exhibit varying bioavailability and may have diverse impacts, even at the same doses.

Given the potential benefits of selenium supplementation and the hazards associated with excessive selenium intake, there is growing interest in exploring the concept of supranutritional selenium supplementation. Broadly speaking, this refers to the administration of selenium supplements with low toxicity and high bioavailability at doses exceeding the normal nutrient level, while causing no harm to organisms, so as to treat or prevent specific diseases effectively. Despite the role of selenium in MASLD having garnered increasing attention recently, a critical review summarizing the effects of supranutritional selenium supplementation on MASLD remains lacking. In consequence, this paper focuses on impacts of the dose and form of selenium supplementation on MASLD and associated hepatic disorders in cell and animal models and epidemiological studies and discusses the crucial points of supranutritional selenium supplementation, which provides valuable insights into potential applications and risks of supranutritional selenium supplementation in MASLD.

## 2. Methods

This review was performed following three steps: conducting a search, reviewing research articles and reviews, and discussing results. The PubMed and Web of Science databases were searched for cell or animal studies and epidemiological research using the two keywords “selenium” and “MASLD”, combining them with other terms such as supranutritional, NAFLD, hepatic diseases, liver diseases, and selenoproteins. To avoid missing any significant relevant papers, reviews on this topic were also reviewed. Among all the retrieved articles, we summarized and synthesized those which deal with impacts of selenium intake (especially supranutritional selenium supplementation) on MASLD and associated liver disorders to integrate this narrative review.

## 3. Occurrence and Development of MASLD

MASLD is a chronic disease with a multifactorial etiology, and its mechanisms have not been entirely explained. The “multiple parallel-hit” hypothesis, which includes various risk factors from dietary, genetic, environmental, and other perspectives, is the most widely accepted hypothesis for MASLD’s etiology [25,26]. These factors include but are not limited to a high-energy diet (e.g., a high-fat diet (HFD) or high-sugar diet), a sedentary lifestyle and a lack of physical activity [27,28], obesity and IR [19], mitochondrial dysfunction and oxidative stress [25], lipotoxicity [29], adipokine dysfunction [30], endocrine disruptors [31], and disturbances in gut microbiota composition [32], as well as specific gene polymorphisms and epigenetic modifications [33]. In medicine, the pathogenesis of MASLD is commonly described as the “two-hit” hypothesis according to the causal relationship between various factors [34]. The “first hit” is caused by many IR-causing disorders, including obesity, diabetes, and hyperlipidemia, and leads to excessive ectopic lipid accumulation in hepatocytes. Then oxidative stress and lipid peroxidation in hepatocytes with excessive lipid deposits contribute to the “second hit”, resulting in liver inflammation, liver fibrosis, and even hepatocellular carcinoma (HCC).

**Figure 1:**
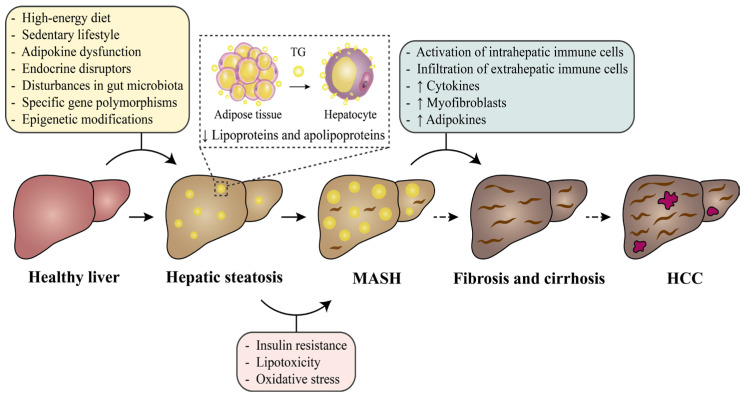
The pathological progression and risk factors for MASLD. Abbreviations: HCC, hepatocellular carcinoma; MASH, metabolic dysfunction-associated steatohepatitis; TG, triglyceride.

Combining the above hypotheses, MASLD progresses via a complicated set of pathological processes, beginning with hepatic steatosis and advancing to metabolic dysfunction-associated steatohepatitis (MASH), eventually leading to hepatic fibrosis, cirrhosis, and/or HCC (Figure 1) [35,36]. A healthy liver is highly susceptible to MASLD when exposed to certain risk factors such as a high-energy diet, a sedentary lifestyle, and so on. The initial manifestation of MASLD is hepatic steatosis, which is characterized by abnormal accumulation of excess TGs in hepatocytes due to reduced adipose tissue storage capacity. Furthermore, the synthesis of lipoproteins and apolipoproteins in hepatocytes is decreased [37], along with impaired mitochondrial function leading to excessive production of reactive oxygen species (ROS). By implementing interventions, such as enhancing physical activity and adopting a balanced diet, hepatic steatosis can be effectively alleviated. Failure to treat hepatic steatosis in time may result in the progression to MASH, driven by IR, lipotoxicity, and oxidative stress [29,38]. As the disease worsens, intrahepatic immune cells such as hepatic stellate cells and dendritic cells become activated, resulting in the infiltration of neutrophils, macrophages, T lymphocytes, and other extrahepatic immune cells into the liver. Simultaneously, cytokines released by immune cells intensify inflammatory responses [39], while hepatic stellate cells gradually differentiate into myofibroblasts [40]. In addition, adipose tissue can secrete adipokines like leptin and adiponectin during inflammatory processes, and their interactions with cytokines may exacerbate MASLD deterioration [30]. If MASH is not treated promptly, advanced stages of MASLD, such as fibrosis, cirrhosis, and HCC, will develop.

In the whole pathological progression of MASLD, both oxidative stress and robust inflammatory responses are critical risk factors. Exogenous nutrients or pharmacological treatments are required to protect hepatocytes from oxidative stress and inhibit adverse inflammatory responses. Selenium is an essential trace element for many organisms and plays a vital role in regulatory and metabolic functions, such as antioxidation and regulation of inflammation. In consequence, selenium is a highly promising exogenous nutrient in the therapy and prevention of MASLD. The mechanisms of selenium regulation in MASLD are described in detail below.

## 4. Mechanisms of Selenium Action in MASLD

**Figure 2:**
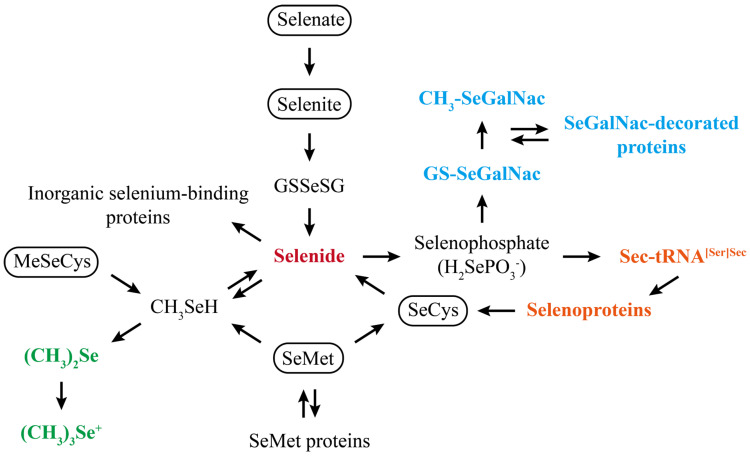
Schematic diagram for the metabolism of selenium. Selenate, selenite, SeMet, SeCys, MeSeCys, and other common dietary forms of selenium gradually transform to selenide (red), which is a precursor for the synthesis of selenoproteins upon intake. Then the central metabolite selenide is used in three major ways: incorporation into selenoproteins (orange), methylation and excretion (green), and conversion to selenosugars (blue). Figure adapted from previous references [41,58,59]. Abbreviations: CH3-SeGalNac, 1β-methylseleno-N-acetyl-D-galactosamine; GS-SeGalNac, 1β-glutathionylseleno-N-acetyl-D-galactosamine; GSSeSG, selenodiglutathione; SeCys, selenocysteine.

The dose and form of selenium supplementation directly or indirectly influence the regulatory effects of selenium in the body. Table 2 summarizes their impacts on MASLD-associated hepatic disorders in experimental animals. Following digestion and absorption, selenium enters the mesenteric venous drainage and is transported to the liver, which serves as the major organ for selenium metabolism. Various forms of selenium are rapidly metabolized into selenide and further used by organisms in three main ways (Figure 2) [41]. Among the several selenium metabolites, selenoproteins play a crucial role in antioxidation and inflammation control. Up to now, a total of 25 selenoproteins have been identified in the human body, encompassing glutathione peroxidase (GPX), thioredoxin reductase (TXNRD), selenoprotein S (SELENOS), selenoprotein M (SELENOM), selenoprotein P (SELENOP), selenoprotein K (SELENOK), selenoprotein W (SELENOW), and selenoprotein I, among others [42,43]. Apart from these known selenoproteins, some novel selenium compounds such as selenoneine may augment the antioxidant activity of the liver and alleviate hepatic injury in the absence of selenoproteins [44].

Most selenoproteins function as antioxidants, and their antioxidant mechanisms are closely associated with ROS. Superoxide anion radicals and hydrogen peroxide are two main types of ROS that come from the mitochondria, endoplasmic reticulum (ER), and NADPH oxidase. ROS can be constantly generated in different hepatocytes as byproducts of energy metabolism [60]. Oxidative stress occurs when the balance between ROS production and clearance is disrupted in favor of the former [61]. In the context of MASLD, excessive lipid accumulation in hepatocytes alters the mechanism of ROS production and results in an overproduction of ROS. Elevated ROS levels in hepatocytes can oxidize and modify various molecules, including DNA and proteins. The accumulation of these damaged cellular components ultimately induces liver damage [62]. In 25 selenoproteins, GPXs and TXNRDs are critical components of the human antioxidant system. GPXs belong to the family of antioxidant enzymes that includes cytosolic GPX (GPX1), gastrointestinal-specific GPX (GPX2), plasma GPX (GPX3), and phospholipid hydroperoxide GPX (GPX4) [63]. The antioxidant mechanism of GPXs involves utilizing glutathione as an electron donor to neutralize intracellular and extracellular hydroperoxides as well as organic peroxides, reducing oxidative stress-induced cellular damage. TXNRDs belong to the flavoprotein family and play a vital role in thioredoxin catalysis as well as the elimination of various ROS. By regulating the activity and redox state of thioredoxin, which is the core regulator of intracellular redox reactions, TXNRDs can maintain ROS homeostasis in cells [64].

Regulating inflammatory responses is another critical function of some selenoproteins. Selenoproteins inhibit the occurrence of inflammation by regulating the expression of inflammatory factors. SELENOS, an ER-resident protein, is induced by the nuclear factor kappa B (NF-κB) pathway in response to ER stress. Suppressing SELENOS with short interfering RNA increased the release of pro-inflammatory factors in macrophages, including interleukin-6 (IL-6) and tumor necrosis factor-α (TNF-α) [65]. Moreover, TNF-α and IL-1β significantly increased the gene expression and protein level of SELENOS in HepG2 cells [66]. These results suggest the existence of a regulatory loop between inflammatory factors and SELENOS, which plays a vital role in mediating inflammation. Additionally, SELENOM is localized in the ER and alleviates metabolic disorders by regulating ER stress and inflammation responses [67]. SELENOM deletion significantly upregulated the expression of pro-inflammatory factors such as Tnf-α and Il-6 in the mouse liver, reduced mitophagy, and aggravated hepatic injury in MASLD [42].

Interestingly, a few selenoproteins impact regulatory pathways exacerbating MASLD. For example, SELENOP, one of the most extensively characterized selenoproteins, has been increasingly associated with the development of IR [68]. SELENOP administration reduced insulin-stimulated phosphorylation of both the insulin receptor and the protein kinase B (Akt) that can impair insulin signaling in hepatocytes, while genetic knockdown of hepatic Selenop1 alleviated both glucose intolerance and IR in mice with type 2 diabetes [69]. In addition, SELENOK promoted the subcellular trafficking of fatty acid translocase (CD36), which could elevate the fatty acid intake by facilitating the recruitment and assembly of coat protein complex II vesicles and eventually increase the distribution of CD36 on the plasma membrane to aggravate hepatic steatosis [70]. Similarly, a recent study showed that SELENOW promoted hepatocyte apoptosis and pyroptosis by regulating metabolic reprogramming to exacerbate the progression of MASLD [71]. Moreover, in cases where the biosynthesis and activity of most selenoproteins reach saturation due to an excessive selenium diet [72], some low-molecular-weight reactive selenium metabolites or intermediates such as selenosugars may exhibit antagonistic effects with TXNRDs and interfere with the related antioxidant system [73]. Based on the above discussion, supranutritional selenium supplementation has a potential positive effect on the prevention and treatment of MASLD, but there are also some risk factors.

## 5. Effects of Dose and Form of Selenium Supplementation on MASLD-Associated Hepatic Disorders

**Figure 3:**
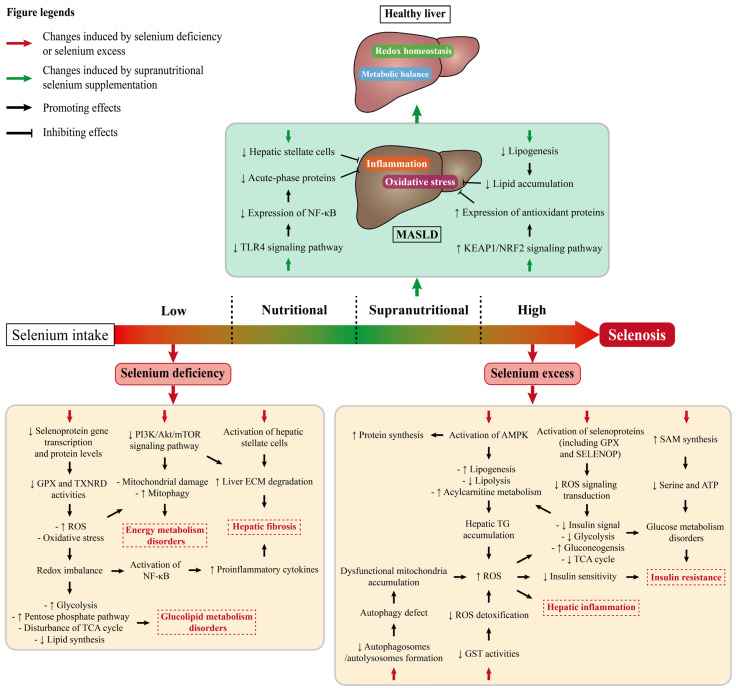
Potential mechanisms of selenium intake in MASLD-associated hepatic metabolism. Abbreviations: Akt, protein kinase B; AMPK, adenosine monophosphate-activated protein kinase; ATP, adenosine triphosphate; ECM, extracellular matrix; GPX, glutathione peroxidase; GST, glutathione-S-transferase; KEAP1, Kelch-like ECH-associated protein 1; mTOR, mammalian target of rapamycin; NF-κB, nuclear factor kappa B; NRF2, nuclear factor erythroid 2-related factor 2; PI3K, phosphatidylinositol 3-kinase; ROS, reactive oxygen species; SAM, S-adenosylmethionine; SELENOP, selenoprotein P; TCA, tricarboxylic acid; TLR4, toll-like receptor 4; TXNRD, thioredoxin reductase.

The beneficial or harmful effects of nutrients depend on the dosage provided. The association between dietary selenium intake and the severity of MASLD-associated hepatic disorders is a U-shaped dose–response relationship. To be more specific, nutritional-to-supranutritional selenium intake can improve the remission and rehabilitation of MASLD; however, insufficient or excessive selenium intake may conversely aggravate MASLD [74]. The underlying mechanisms of this dose–response relationship are shown in Figure 3. Moreover, selenium is classified as inorganic selenium, organic selenium, SeNPs, and other forms based on its structure and composition. Different forms of selenium exhibit varying biological activities and bioavailability, resulting in various impacts on MASLD-associated hepatic disorders.

Dietary selenium deficiency has been demonstrated to be associated with the development of Keshan disease, Kaschin–Beck disease, cancer, cardiovascular disease, etc. Insufficient selenoprotein synthesis is the main effect of selenium deficiency. In rat models, selenium deficiency caused growth delay and decreased tissue selenium concentrations, antioxidant activities, and selenoproteins expression [75]. To investigate the mechanisms underlying liver pathology caused by selenium deficiency, a comprehensive analysis of the hepatic metabolome, lipidome, global proteome, and whole transcriptome was conducted in pure-line pig models fed a selenium-deficient diet [9]. The results showed that selenium deficiency induced redox imbalance, which disrupted glucolipid metabolism through downregulation of selenoprotein expression and impairment of glutathione antioxidant system function [9]. At the same time, the central carbon metabolism of the liver was reprogrammed and the NF-κB signaling pathway was activated, resulting in liver inflammation [9]. In rats, long-term dietary selenium deficiency induced liver damage by altering hepatocyte ultrastructure and expression of metalloproteinase (MMP) and tissue inhibitors of MMPs [76]. MMPs are secreted by activated hepatic stellate cells, resulting in extracellular matrix breakdown and liver fibrosis. Similarly, short-term dietary selenium deficiency could inhibit the Akt/mammalian target of rapamycin (mTOR) signaling pathway and cause liver fibrosis [77]. Another study revealed that selenium deficiency induced liver fibrosis with the occurrence of mitophagy, which was related to the mTOR signaling pathway [55]. In non-mammals such as chickens, selenium deficiency triggered oxidative stress, ER stress, and apoptosis in the liver via the potential involvement of oxidative–ER stress pathways [46]. In conclusion, selenium deficiency can accelerate MASLD progression by affecting specific signaling pathways, inhibiting antioxidant systems and exacerbating inflammatory responses.

As the dose increases, the benefits of selenium supplementation for MASLD-associated hepatic disorders become apparent, especially at supranutritional levels. In fact, the exact dosage of supranutritional selenium supplementation depends on different experimental models. A transcriptome study on selenium toxicity indicated that the dose range of supranutritional selenium supplementation in rodents was 8−20 times higher than the normal requirements [78], but some other studies suggested a lower dose. For example, the supranutritional supplementation of sodium selenite at 1.0 mg Se/kg diet (the dietary selenium content of the control group was 0.3 mg Se/kg diet) may be the optimum concentration against fat deposition and liver damage induced by HFD in pig models with MASLD [38]. Significantly, inorganic selenium, represented by sodium selenite and sodium selenate, possesses a significant capacity for selenium accumulation in the liver and kidneys, inducing toxicity even at relatively low doses (Table 3) [79,80,81]. Therefore, the supplementing dosage of it must be tightly controlled. Organic selenium is another regular selenium supplement. SeMet, selenocystine, and methylselenocysteine could ameliorate MASLD through 5-hydroxytryptophan/bile acid enterohepatic circulation in mice with MASLD [48]. Supranutritional supplementation with selenium-enriched (Se-enriched) spirulina at more than four times the dietary selenium content of the control group effectively ameliorated HFD-induced hepatic injury and IR by inhibiting lipogenesis gene expression and hepatic lipid accumulation, as well as improving oxidative stress in hepatocytes by promoting the Kelch-like ECH-associated protein 1/nuclear factor erythroid 2-related factor 2 pathway [49]. Selenium-containing tea polysaccharides from a new variety of Se-enriched Ziyang green tea were reported to protect mice from high-fructose-induced IR and hepatic oxidative injury, particularly at supranutritional doses [50]. Furthermore, selenoneine, a novel organic selenium-containing compound isolated from various tissues of tuna, has been found to effectively alleviate hepatocellular injury and hepatic steatosis [82]. Selenoneine feeding in MASLD mice lacking the farnesoid X receptor increased hepatic antioxidant activity while decreasing hepatic lipid production [44]. Nonetheless, unlike other forms of selenium, selenoneine did not increase hepatic selenoprotein expressions but reduced mRNA expression levels of Gpx1 and Selenop in the liver [44]. It is speculated that selenoneine does not indirectly regulate redox processes in the liver through selenoproteins. The mitigating effects of selenoneine on MASLD may depend on its unique chemical structure and synergistic interaction with active ingredients from the same food source, such as docosahexaenoic acid and eicosapentaenoic acid.

Apart from supplementing inorganic and organic non-nano-selenocompounds, more research has been conducted on the preparation, characterization, and application of SeNPs. SeNPs demonstrate lower toxicity (Table 3) and display superior anti-free radical, anti-inflammatory, anticancer, and antibacterial properties than other forms of selenium [88]. SeNP administration at supranutritional levels (1.0 and 0.8 mg Se/kg body weight (BW)) exhibited beneficial effects in attenuating microplastic-induced MASLD [57] and improving hepatic antioxidant capacity [54], respectively. Novel SeNPs synthesized by *Lactobacillus coryniformis* ES23 alleviated MASH via modulating lipid metabolism and ferrous ion homeostasis [89]. Amorphous selenium nanodots (A SeNDs) at 0.3 mg Se/kg BW can activate vascular endothelial growth factor (VEGF) receptor 1 and then inhibit phosphorylation of the c-Jun *N*-terminal kinase/p38 signaling pathway [56]. This led to a reduction in hepatocyte steatosis, oxidative stress, and inflammatory reactions while improving hepatic structure and liver function in Sprague-Dawley rats with MASLD [56]. Apart from SeNPs, a specialized study investigated the presence of facultative protein selenation, which was initiated by increasing cellular levels of organic selenium [90]. This phenomenon may be associated with the regulation of redox sensitivity and adipose tissue thermogenesis. Moreover, it was found that this process protected mice from obesity by enhancing thermogenesis without inducing liver inflammation or fibrosis [90]. These newly developed or recently discovered forms of selenium are expected to provide additional sources for supranutritional selenium supplementation, potentially replacing frequently used selenium supplements such as sodium selenite and SeMet.

However, a supranutritional or excessive selenium diet is a potential etiological factor of IR which may stimulate hepatic de novo lipogenesis and gluconeogenesis, as well as increase TG accumulation and oxidative damage in the liver, all of which lead to hepatic IR [91]. Moreover, excessive selenium excretion accompanied by metabolic reprogramming of S-adenosylmethionine and serine synthesis induces glucose metabolism disorders [92]. For instance, selenium intake up to eight times the requirement (in the form of Se-enriched milk casein) impaired hepatic insulin sensitivity via downregulating the expression of insulin receptor substrate, phosphatidylinositol 3-kinase, and Akt [51]. Furthermore, compared with a 0.3 mg Se/kg diet, pigs fed with a 3.0 mg Se/kg diet in the form of Se-enriched yeast exhibited hepatic lipid accumulation [52]. This effect may be associated with the stimulation of lipogenesis and gluconeogenesis, as well as the suppression of lipolysis [52]. A similar study found that fatty acid accumulation in pig livers was related to increased acylcarnitine metabolism when diets were supplemented with SeMet at 2.5 mg Se/kg [47]. Therefore, we should treat supranutritional selenium supplementation with caution.

Once the intake of selenium exceeds the body’s tolerance level, selenosis will appear. Many animal studies have demonstrated that the liver is the main target organ of selenosis [93]. Selenium oversupply may exacerbate MASLD-associated hepatic disorders by impairing hepatic functions. To be more specific, excessive inorganic selenium exposure promotes hepatic redox imbalance and ROS-induced DNA damage [94]. Moreover, the toxicity of excess organic selenium is also related to the inactivation and aggregation of proteins [95]. Evidence from animal models under pathological conditions showed that excessive selenium intake had detrimental effects on liver health. In diabetic mouse models, long-term excessive supplementation with selenate at a dose approximately 16.8 times higher than the recommended dietary intake resulted in increased insulin production and secretion [45]. By upregulating lipogenic and inflammatory genes while downregulating genes associated with detoxification, antioxidant defense capacity was reduced, eventually exacerbating fatty liver degeneration [45]. In non-mammalian models, excess L-SeMet caused selenium stress to affect liver development and glucolipid metabolism by inhibiting lipolysis, glycolysis, and autophagy in zebrafish embryos [96], and a dietary selenium level of 4.21 mg/kg with Se-enriched yeast was found to be toxic for on-growing grass carp [97]. Under physiological conditions, excessive dietary selenium supplementation may alter hepatic metabolism and increase risk factors related to the initiation and progression of MASLD. Three microarray studies of transcripts in rodents revealed that selenium toxicity (5 mg Se/kg diet) vastly affected the liver transcriptome, whereas selenium deficiency or supranutritional selenium intake elicited relatively minimal changes [78]. A gene set enrichment analysis compared liver transcript expressions between rats and turkeys under high-selenium (5 mg Se/kg diet) conditions [98]. Surprisingly, no common gene sets were found to be significantly and consistently regulated by a high-selenium diet in either species, suggesting the presence of species-specific differences in selenosis [98].

To summarize, the effects of the dose and form of selenium supplementation on MASLD-associated hepatic disorders seem to be independent but interconnected. On the one hand, the dose of selenium supplementation determines the body’s selenium homeostasis and regulatory mechanisms for MASLD. On the other hand, the form of selenium supplementation influences both the beneficial dosage range of selenium in MASLD and the functional forms of selenium in the body. To improve MASLD, supranutritional selenium supplementation must be administered with an appropriate dose and a superior form.

## 6. Results of Epidemiological Studies on Selenium and MASLD

### 6.1. Associations Between In Vivo Selenium Levels and MASLD

Numerous animal studies have consistently demonstrated that selenium supplementation plays a critical role in the pathogenesis and progression of MASLD. For populations, epidemiological studies investigating the relationship between in vivo selenium levels and the risk of MASLD are also receiving increasing attention. Nevertheless, due to complex factors such as regional variations, racial disparities, and dietary diversity, these studies sometimes yield inconsistent or even contradictory conclusions.

The following studies have indicated a potential inverse association between in vivo selenium levels and the risk of MASLD, evidenced by lower selenium levels in patients with MASLD. For example, one bioinformatic analysis compared gene expression levels of known selenoproteins and selenium-containing pathways between healthy individuals and MASLD patients from online repositories, suggesting that the liver with MASLD may have lower selenium levels than the healthy liver [99]. A serum untargeted metabolomic analysis from Hubei Province in China showed that high-selenium exposure was negatively associated with serum lipid profiles and decreased the risk of dyslipidemia [100]. In advanced MASLD, some cross-sectional studies demonstrated a decline in serum selenium levels corresponding to an increase in the severity of liver cirrhosis [101,102]. Additionally, a strong association between high selenium status and a lower risk of HCC was observed in the European Prospective Investigation into Cancer and Nutrition study [103]. Specifically, concentrations of serum selenium and SELENOP were significantly lower in HCC cases than in controls (geometric means: 71.3 compared with 85.2 μg/L and 4.3 compared with 5.4 mg/L, respectively) [103]. Similarly, in 29 HCC subjects from Austria, selenium levels and GPX4 expression were inversely correlated with serum levels of VEGF and IL-8 (two clinically significant indicators of HCC), as well as tumor size [104]. These findings suggest that reduced selenium levels may exacerbate the severity of MASLD through disturbing relative gene expression and cytokine secretion. Furthermore, a randomized controlled trial involving 82 cirrhosis subjects discovered that moderate-to-severe cirrhosis may cause mild functional selenium deficiency associated with impaired SeMet metabolism, and the incidence of selenium deficiency increased as cirrhosis worsened [105]. Therefore, selenium supplementation at supranutritional levels can be considered as a strategy for prevention or therapy in MASLD patients, particularly those in advanced stages with low selenium status.

Controversially, certain cross-sectional studies have revealed non-linear or even positive associations between in vivo selenium levels and the prevalence of MASLD. In a cross-sectional analysis of 3827 American adults from the National Health and Nutrition Examination Survey (NHANES) (2011–2016), positive correlations between serum selenium levels and both serum alanine aminotransferase (ALT) activity and suspected prevalence of MASLD were found above the serum selenium level >130 μg/L, while no associations were observed below 130 μg/L [106]. Another cross-sectional study that was conducted between 2017 and 2019 on healthy populations involved 137 Italian adults to investigate the relationships between serum ALT levels and total serum selenium, as well as urinary selenium levels [107]. The results revealed that urinary selenium levels and a few chemical selenium forms in serum were positively correlated with ALT, but total serum selenium exhibited an inverse association with ALT below 120 μg/L [107]. Additionally, in a cross-sectional study conducted in Shanghai, a total of 8550 subjects aged 40 years and older were included to explore the association between plasma selenium concentration and MASLD prevalence [108]. The results showed that the median concentration of plasma selenium in all participants was 213 μg/L, and participants with MASLD had elevated plasma selenium levels compared with those without MASLD (medians: 270.2 μg/L and 192.5 μg/L, respectively) [108]. Interestingly, a large-sample, cross-sectional study based on NHANES (2017–2018) found that blood selenium levels (>205.32 and ≤453.62 μg/L) were positively associated with MASLD, whereas participants with lower blood selenium levels showed a higher percentage of advanced liver fibrosis [109]. These findings further confirm that patients in the middle or advanced stages of MASLD are more likely to require supranutritional selenium supplementation. More significantly, further research is urgently required to determine whether fluctuation in selenium levels is the cause or the consequence of MASLD.

### 6.2. Associations Between Dietary Selenium Intake and MASLD

Based on the above discussion, dietary selenium intake as a vital factor affecting selenium levels appears to be closely related to the occurrence and development of MASLD. A cross-sectional analysis based on the 2004 and 2015 Canadian Community Health Surveys revealed a negative correlation between the intake of selenium and liver fat in females [110]. Nevertheless, a cross-sectional study from China observed a positive association between dietary selenium intake (mean: 42.3 μg/day) and the prevalence of MASLD in middle-aged and elderly individuals [111]. Another study in Iran reported a slight positive association between dietary selenium intake (mean: 109.29 μg/day) and the risk of MASLD [112]. Additional evidence is provided by a cross-sectional study in a healthy Italian population that revealed that the association of dietary selenium intake with the level of ALT was weakly positive up to 100 μg/day but null above that value [107]. It is interesting that a meta-analysis conducted in patients with chronic liver diseases showed that correlations between selenium intake and the risk of MASLD were conflicting with significant heterogeneity [113]. Regarding advanced MASLD, a high dietary selenium intake below 400 μg/day tended to be a protective factor [113].

In fact, precise dietary selenium intake is difficult to evaluate, which influences the confidence of related studies to some extent. It remains unclear whether selenium homeostasis disorders or dietary selenium intake affects selenium levels in patients with MASLD. Furthermore, most research consists of cross-sectional studies that cannot establish a causal relationship between dietary selenium intake and MASLD. Interventional trials investigating the effect of supranutritional selenium supplementation on MASLD are expected to be performed urgently. The clinical study designs are similar to those of some reported randomized controlled trials regarding selenium supply [114,115,116]. Firstly, enough participants diagnosed with MASLD are recruited. Secondly, these participants are randomly assigned to one of three groups for treatment (6–12 months): placebo, “nutritional” (the recommended nutrient intake (RNI) of selenium), or “supranutritional” (2–4 times the RNI of selenium). Finally, the physical conditions (e.g., body mass index, serum lipid level, serum ALT level, and total serum selenium) of all participants are evaluated every two months. All investigators and participants remain blinded to randomization status until the conclusion of the trial.

## 7. Crucial Points Regarding Supranutritional Selenium Supplementation in MASLD

### 7.1. Accurate Assessment of Selenium Nutritional Status in MASLD Patients

Because of the potential risks associated with supranutritional selenium supplementation (such as IR and hepatic lipid deposition) and the complex pathological mechanisms involved in MASLD, not all patients with MASLD are recommended for supranutritional selenium supplementation. Accurate assessment of selenium nutritional status in MASLD patients is essential before supranutritional selenium supplementation. Currently, SELENOP and GPXs have emerged as the primary biomarkers used to reflect selenium nutritional status, particularly liver-derived SELENOP and kidney-derived GPX3 [117]. SELENOP is mainly synthesized and secreted in hepatocytes, acting as the principal selenium transporter from the liver to other tissues, and accordingly represents the predominant physiological form of selenium transport [118]. Serum SELENOP is tightly correlated with selenium status in patients with low-to-moderate dietary selenium intake [119]. When excessive selenium supplementation is administered, SELENOP is also an early indicator of selenosis to some extent. In a clinical trial involving therapeutic selenite doses, plasma SELENOP acted as a reliable biomarker for monitoring therapeutic selenium administration even if it exceeded the recommended upper intake level (10 mg SELENOP/L) [120]. Furthermore, GPX activities are closely related to patients’ selenium nutritional status and reflect overall antioxidant levels. GPX3, as a crucial selenoprotein in mammals depending on SELENOP-mediated selenium transport, is initially synthesized by the kidneys and subsequently secreted into the bloodstream. Nowadays, GPX3 activities have been applied to diagnose and evaluate selenium nutrition in many clinical studies [105,117,121].

Moreover, some novel selenium biomarkers are emerging. For example, SELENOP autoantibodies were identified in Hashimoto’s thyroiditis, and their titers were conversely associated with GPX3 activities via their impairment of SELENOP-dependent selenium transport, reflecting extra information about health risks related to selenium deficiency [122]. According to Figure 2, selenium is metabolized into methylated metabolites and then excreted through urine or breath. Some mass spectrometry studies have identified that monomethylated selenium metabolites (selenosugars) can be used as specific indices within a low-toxicity range [123,124]. With high selenium supplementation, increased selenosugar formation occurs and further increases selenosugar-decorated proteins. Interestingly, the species and proportions of selenium metabolites, including selenosugars, are closely related to the form of selenium supply [59,95,125]. In addition to the abovementioned traditional and novel selenium biomarkers, incorporating selenium parameters (such as selenium concentrations in total blood, serum, plasma, nails, or hair) can allow the obtainment of a more comprehensive assessment of selenium nutritional status in patients with MASLD.

### 7.2. Development of Novel Functional Selenium Forms Like SeNPs for MASLD

The continuous emergence of novel functional selenium forms enables the widespread application of supranutritional selenium supplementation. With the synergistic integration of fundamental selenium research, nanotechnology, and biotechnology, SeNPs have shown great potential as a promising source of selenium in various fields, such as food and medicine [126]. Physical, chemical, and biological methods can be used to transform inorganic selenium into SeNPs, so as to reduce toxicity and increase bioavailability. Among them, biological transformation, especially microbial transformation, is an eco-friendlier method. Considering the potent biological functions of some beneficial microorganisms, exploring strategies to activate the synergistic effect (“1 + 1 > 2”) of Se-enriched microorganisms (SeMs), including SeNPs and microorganisms, is a highly valuable avenue for research. Table 4 summarizes several SeNPs and SeMs from diverse microbial systems. Furthermore, some other studies have revealed that the amorphous structure of biogenic SeNPs determines their bioavailability [127].

Except for SeNPs, some novel selenium-containing proteins and polysaccharides are also prospective forms of selenium to treat MASLD and associated diseases. For example, seleno-ovalbumin, a selenium-conjugating protein, was formed by combining ovalbumin with inorganic selenium, which induced ROS-dependent mitochondrial-mediated apoptosis in HepG2 cells [141]. Similarly, seleno-β-lactoglobulin, which may cause apoptosis and autophagy in HCC cell lines, was synthesized by combining β-lactoglobulin with selenium dioxide [142,143]. Many selenium-containing polysaccharides have remarkable antioxidant and biological activities after biological transformation. For example, Se-enriched *Pleurotus ostreatus* polysaccharide extracted from fresh fruiting bodies of *P. ostreatus* using selenite sodium displayed potent in vitro antioxidant capacity [144]. Another Se-polysaccharide was isolated from fruiting bodies of Se-enriched *Grifola frondosa* and exhibited a superior ability to scavenge free radicals compared to *G. frondosa* polysaccharide [145]. However, the restriction is the lack of in vivo experimental evidence supporting the antioxidation, anticancer, and other capabilities of the above selenium forms. Additionally, combining these newly developed forms of selenium with other functional ingredients (such as silibinin [146], puerarin [147], magnesium [148], zinc [149], vitamin E [150], etc.) may better improve MASLD.

## 8. Conclusions and Future Directions

Selenium is an essential trace element with a lot of unknowns in MASLD. Selenium supply can ameliorate oxidative stress and mitigate excessive inflammatory responses during the onset and progression of MASLD. Nevertheless, it is important to note that normal nutritional selenium intake is usually insufficient for organisms under pathological conditions, while excessive selenium intake can directly lead to liver injury and metabolic disorders. Therefore, studies on supranutritional selenium supplementation for MASLD have progressed but with differing opinions. In this review, we are the first to comprehensively discuss potential applications and risks of supranutritional selenium supplementation in MASLD based on a large number of cell, animal, and epidemiological studies, and ultimately come to the following conclusions:

(1) Based on the effects of different doses and forms of selenium on MASLD-associated hepatic disorders, supranutritional selenium supplementation must establish a tolerable dosage range according to the severity of MASLD and select superior forms of selenium, such as organic selenium and SeNPs.
(2) Numerous epidemiological studies have observed that hepatic fibrosis, cirrhosis, and HCC (the middle or late stages of MASLD) are more prone to selenium functional deficiencies, indicating that patients in these stages are ideal candidates for supranutritional selenium supplementation. Meanwhile, MASLD patients who live in high-selenium areas or who have a history of diabetes, hyperglycemia, hyperinsulinemia, etc., should exercise caution regarding supranutritional selenium supplementation.
(3) Determining selenium nutritional status is a prerequisite for the utilization of supranutritional selenium supplementation in MASLD. Furthermore, novel forms of selenium with enhanced functionality may facilitate broader adoption of supranutritional selenium supplementation.

Concerning the future, it is worth noting that studies on supranutritional selenium supplementation and MASLD still face many challenges and bottlenecks. For example, in most animal experiments, doses of selenium supplementation are typically determined according to animals’ daily food intake or BW (Table 2), which may cause inconsistent dose units and introduce bias when comparing doses across different studies. The European Food Safety Authority has proposed default factors for converting chemical substance concentrations in feed or drinking water into daily doses for experimental animal studies, but these default factors are also required to be adjusted according to specific conditions [151]. Different methods of selenium intervention, such as oral feed, gavage, or intraperitoneal injection, directly affect the absorption and metabolism of selenium in animals, which influences the final results. Moreover, epidemiological studies on selenium and MASLD primarily consist of cross-sectional studies which are limited to specific regions or populations and unable to establish causal relationships. Relationships between selenium homeostasis disorders and the occurrence and development of MASLD have not been fully elucidated. Relevant interventional trials are expected to be performed urgently. At the same time, studies on selenosis remain limited. The construction of various selenosis models and the complete elucidation of the underlying mechanisms of selenosis can be expected to provide more valuable evidence regarding the potential applications and risks of supranutritional selenium supplementation in MASLD.

## Figures and Tables

**Table 1:** Dietary reference intake of selenium for adults.

Country or Body	RNI (μg/Day)	UL (μg/Day)	Ref.
WHO	26 (males), 34 (females)	400	[16]
China	60	400	[20]
USA	55	400	[16]
EFSA	70 (AI)	255	[21,22]
Japan	30–35 (males), 25 (females)	450 (males), 350 (females)	[23]
Korea	60	400	[24]

Abbreviations: AI, adequate intake; EFSA, European Food Safety Authority; RNI, recommended nutrient intake; UL, tolerable upper intake level.

**Table 2:** Animal studies on the impacts of selenium supplementation on MASLD-associated hepatic disorders.

Form	Dose	Experimental Animal	Experimental Period	Route of Administration	Main Effects	Ref.
Inorganic selenium						
Sodium selenate	0.8 mg sodium selenate/kg BW	Diabetic db/db mice	9 weeks	Gavage	Increased insulin production and secretion but reduced antioxidant defense capacity, which exacerbated fatty liver degeneration.	[45]
Sodium selenite	0.3, 1.0 and 3.0 mg Se/kg diet	Pigs	16 weeks	Oral feed	The supplementation of selenium at 1.0 mg/kg may be the optimum concentration against liver damage induced by HFD.	[38]
Sodium selenite	0.033 and 0.2 mg Se/kg diet	Chickens	15, 25, 35, 45, 55 or 65 days	Oral feed	Selenium deficiency induced oxidative stress, ER stress, and apoptosis in chicken livers.	[46]
Organic selenium						
SeMet	0.25 and 2.5 mg Se/kg diet	Pigs	60 days	Oral feed	High SeMet intake resulted in hyperglycemia, hyperinsulinemia, and fatty acid accumulation in the liver.	[47]
SeMet	0.3 mg Se/kg diet	Pigs	16 weeks	Oral feed	Improved the redox imbalance, which led to hepatic metabolic reprogramming and inflammation in selenium deficiency.	[9]
Selenocystine, SeMet, and MeSeCys	0.25 and 0.5 mg Se/kg BW	C57BL/6 mice	16 weeks	Gavage	Improved MASLD in mice through 5-hydroxytryptophan/bile acid enterohepatic circulation.	[48]
Se-enriched spirulina	0.45 mg Se/kg diet	C57BL/6 mice	12 weeks	Oral feed	Improved the hepatic injury and IR in HFD mice.	[49]
Se-GTP	200, 400 and 800 mg Se-GTP/kg BW	Kunming mice	8 weeks	Gavage	Ameliorated the high fructose-induced IR and hepatic oxidative injury, which was more effective at a high dose.	[50]
Se-enriched milk casein	0.25, 0.5 and 2.0 mg Se/kg diet	SD rats	7 weeks	Oral feed	Supranutritional selenium intake up to 8 times the requirement had similar negative effects on hepatic insulin sensitivity as consuming an HFD.	[51]
Se-enriched yeast	0.3 and 3.0 mg Se/kg diet	Pigs	11 weeks	Oral feed	High dietary selenium intake altered lipid metabolism and protein synthesis in liver and muscle of pigs.	[52]
Se-enriched yeast	0.15 and 0.65 mg Se/kg diet	Goats	10 weeks	Oral feed	Supranutritional selenium alleviated hepatic oxidative and inflammatory lesions induced by a high-concentrate diet.	[53]
Other forms						
Selenoneine	0.3 mg Se/kg diet	Fxr-null mice	4 months	Oral feed	Attenuated hepatic steatosis and hepatocellular injury in an MASLD mouse model.	[44]
SeNPs	0.2, 0.4 and 0.8 mg Se/kg BW	SD rats	2 weeks	Gavage	Improved hepatic antioxidant capacity at supranutritional levels.	[54]
Chondroitin sulfate SeNPs	0.1 and 0.2 mg Se/kg diet	SD rats	12 weeks	Oral feed	Prevented liver fibrosis, maintained normal energy metabolic activity, and decreased mitophagy.	[55]
A SeNDs	0.3 mg Se/kg BW	SD rats	8 weeks	Gavage	Reduced hepatocyte steatosis, oxidative stress, and inflammatory reactions and improved hepatic structure and liver function in MASLD rats.	[56]
SeNPs	1 mg Se/kg BW	Kunming mice	4 weeks	Gavage	Attenuated liver lipid accumulation and degeneration caused by polystyrene microplastics.	[57]

Abbreviations: A SeNDs, amorphous selenium nanodots; BW, body weight; ER, endoplasmic reticulum; Fxr, farnesoid X receptor; HFD, high-fat diet; IR, insulin resistance; MASLD, metabolic dysfunction-associated steatotic liver disease; MeSeCys, methylselenocysteine; SD, Sprague-Dawley; Se-enriched, selenium-enriched; Se-GTP, selenium-containing tea polysaccharides; SeMet, selenomethionine; SeNPs, selenium nanoparticles.

**Table 3:** Toxicity studies of SeNPs in experimental animals.

Size of SeNPs (nm)	Source of SeNPs	Form of Selenium in the Control Group	Dose Gradient	Experimental Animals	Main Conclusions	Ref.
20–60	Chemical synthesis	Sodium selenite	2, 4, and 6 mg Se/kg BW	Kunming mice	A high dose of sodium selenite caused more pronounced oxidative stress, greater liver injury, and prominent retardation of growth than SeNPs.	[83]
20–60	Chemical synthesis	SeMet	5 and 10 mg Se/kg BW	Kunming mice	SeNPs functioned as antioxidants with a reduced risk of toxicity and a comparable ability to increase selenoenzymes to SeMet.	[84]
20–60	Chemical synthesis	MeSeCys	5 and 10 mg Se/kg BW	Kunming mice	SeNPs could serve as potential chemopreventive agents with reduced risk of toxicity compared to MeSeCys.	[85]
20–60	Chemical synthesis	Sodium selenite and high-selenium protein	2, 3, 4, and 5 mg Se/kg diet	SD rats	SeNPs were less toxic than selenite and high-selenium protein in the 13-week rat study.	[80]
70–90	Chemical synthesis	Sodium selenite	1 and 4 mg Se/kg BW	Swiss albino mice	SeNPs at low doses exhibited antioxidant effects in the liver compared to the high dose of SeNPs and the high and low doses of sodium selenite.	[81]
80–220	Biosynthesis	Selenium dioxide	2.5, 5, 10, and 20 mg Se/kg BW	NMRI mice	The biogenic SeNPs were much less (26-fold) toxic than selenium dioxide, and a dose of 20 mg Se/kg BW was accompanied by signs of toxicity.	[86]
100–500	Biosynthesis	Sodium selenate, sodium hydroselenite, selenoaminoacids, and Se-enriched yogurt powder	0.5, 5, and 50 mg Se/kg diet	BDF1 mice	The toxicity of selenium species decreased in the following order: selenate > selenite > SeNPs > selenoaminoacids > Se-enriched yogurt powder.	[87]

**Table 4:** Characteristics of microbial-derived SeNPs and SeMs.

Microorganisms	Precursor of SeNPs	Size of SeNPs (nm)	Functions of SeNPs and SeMs	Ref.
*Bacillus cereus* YC-3	Sodium selenite	116.87 ± 35.45	Antioxidant and anti-apoptotic activities (SeNPs); attenuate liver lipid accumulation and degeneration (SeNPs).	[57,128]
*Lactobacillus coryniformis* ES23	Sodium selenite	127.4 ± 41.2	Alleviate MASH (SeNPs).	[89]
*Lactobacillus casei* ATCC 393	Sodium selenite	50–80	Anticancer and antioxidant activities (SeNPs); alleviate H_2_O_2_-induced intestinal epithelial barrier dysfunction (SeNPs); alleviate intestinal barrier dysfunction induced by deoxynivalenol (SeNPs and SeMs).	[129,130,131,132]
*Lactobacillus acidophilus* HN23	Sodium selenite	60–300	Reduce hepatocyte lipid deposition and oxidative damage (SeNPs).	[133]
*Bifidobacterium animalis* H15	Sodium selenite	40–200	Alleviate dextran sulfate sodium-induced colitis (SeNPs).	[134]
*Penicillium tardochrysogenum* OR059437	Sodium selenate	82.31 ± 22.10	Antioxidant, antimicrobial, and anticancer activities (SeNPs).	[135]
*Streptomyces parvulus* MAR4	Sodium selenate	48.8–129.0	Antimicrobial and anticancer activities (SeNPs).	[136]
*Rahnella aquatilis* HX2	Sodium selenite	193–513	Anticancer activity (SeNPs).	[137]
*Lactobacillus casei* ATCC 393	Sodium biselenite	170–550	Inhibit colon cancer cell growth in vitro and in vivo (SeNPs and SeMs).	[138]
*Lacticaseibacillus rhamnosus* SHA113	Sodium selenite	42.4 ± 10.5	Protect the liver and intestinal tract from injury by lead (SeMs).	[139]
*Levilactobacillus brevis* 23017	Sodium selenite	50–80	Improve the immune effect of the alum adjuvant vaccine (SeMs).	[140]

Abbreviations: SeMs, Se-enriched microorganisms.

## Data Availability

Data availability is not applicable to this article as no new data were created or analyzed in this study. Copyright Statement: The copyrights of tables and figures in the paper belong to the authors of this review.

## Abbreviations

A SeNDs	Amorphous selenium nanodots
AI	Adequate intake
Akt	Protein kinase B
ALT	Alanine aminotransferase
AMPK	Adenosine monophosphate-activated protein kinase
ATP	Adenosine triphosphate
BW	Body weight
CD36	Fatty acid translocase
CH_3_-SeGalNac	1β-methylseleno-N-acetyl-D-galactosamine
ECM	Extracellular matrix
EFSA	European Food Safety Authority
ER	Endoplasmic reticulum
Fxr	Farnesoid X receptor
GPX	Glutathione peroxidase
GSSeSG	Selenodiglutathione
GS-SeGalNac	1β-glutathionylseleno-N-acetyl-D-galactosamine
GST	Glutathione-S-transferase
HCC	Hepatocellular carcinoma
HFD	High-fat diet
IL	Interleukin
IR	Insulin resistance
KEAP1	Kelch-like ECH-associated protein 1
MASH	Metabolic dysfunction-associated steatohepatitis
MASLD	Metabolic dysfunction-associated steatotic liver disease
MeSeCys	Methylselenocysteine
MMPs	Metalloproteinases
mTOR	Mammalian target of rapamycin
NAFLD	Non-alcoholic fatty liver disease
NF-κB	Nuclear factor kappa B
NHANES	National Health and Nutrition Examination Survey
NRF2	Nuclear factor erythroid 2-related factor 2
PI3K	Phosphatidylinositol 3-kinase
RNI	Recommended nutrient intake
ROS	Reactive oxygen species
SAM	S-adenosylmethionine
SD	Sprague-Dawley
SeCys	Selenocysteine
Se-enriched	Selenium-enriched
Se-GTP	Selenium-containing tea polysaccharide
SELENOK	Selenoprotein K
SELENOM	Selenoprotein M
SELENOP	Selenoprotein P
SELENOS	Selenoprotein S
SELENOW	Selenoprotein W
SeMet	Selenomethionine
SeMs	Se-enriched microorganisms
SeNPs	Selenium nanoparticles
TCA	Tricarboxylic acid
TGs	Triglycerides
TLR4	Toll-like receptor 4
TNF	Tumor necrosis factor
TXNRD	Thioredoxin reductase
UL	Tolerable upper intake level
VEGF	Vascular endothelial growth factor
